# Can American Joint Committee on Cancer prognostic groups be individualized in patients undergoing surgery for Stage IV invasive upper tract Urothelial Carcinoma?

**DOI:** 10.7150/jca.50417

**Published:** 2021-02-02

**Authors:** Zaishang Li, Xueying Li, Ying Liu, Jiequn Fang, Xueqi Zhang, Kefeng Xiao

**Affiliations:** 1 Department of Urology, Shenzhen People's Hospital, The Second Clinic Medical College of Jinan University 518060, Shenzhen, Guangdong, P. R. China.; 2 Department of Urology, First Affiliated Hospital of Southern University of Science and Technology, 518060, Shenzhen, Guangdong, P. R. China.; 3 Department of Urology, Minimally Invasive Urology of Shenzhen Research and Development Center of Medical Engineering and Technology, 518060, Shenzhen, Guangdong, P. R. China.; 4 Department of Oncology, The Seventh Affiliated Hospital Sun Yat-sen University, 518107, Shenzhen, Guangdong, P. R. China.

**Keywords:** urothelial carcinoma, upper urinary tract, tumor-node-metastasis, mortality, survival

## Abstract

**Purpose:** We explored whether the modified American Joint Committee on Cancer tumor-node-metastasis prognostic stage group IV can be individualized in a large population-based cohort of surgically treated invasive upper tract urothelial carcinoma (UTUC) patients.

**Methods:** Invasive UTUC patients from the Surveillance, Epidemiology and End Results database (2004-2015) were screened for inclusion. A total of 10,482 eligible cases were identified. Cancer-specific survival (CSS) after surgery was analyzed using Kaplan-Meier plots.

**Results:** According to the most recent pathological prognostic group classification, the 5-year mortality rates of T4NxM0 (n=493), TxN1M0 (n=597), TxN2M0 (n=424) and pTxNxM1 (n=677) patients were 41.1% (95% CI 35.2% to 47.0%), 38.6% (95% CI 33.1% to 44.1%), 40.4% (95% CI 33.0% to 47.8%) and 14.2% (95% CI 9.9% to 18.5%), respectively (T4N0M0 *vs.* TxNxM1, *P*<0.001; TxN1M0 *vs.* TxNxM1, *P*<0.001; TxN2M0 *vs.* TxNxM1, *P*<0.001). Stage IV tumors were subdivided on the basis of the mortality data (Modification 1): stage IVa tumors were considered nonmetastatic (T4NxM0, TxN1-2M0; 5-year CSS 39.9%), and stage IVb tumors were considered metastatic (pTxNxM1; 5-year CSS 14.2%). Stage IV tumors were also subdivided according to the grade classification (Modification 2): stage IVa tumors were considered low grade (T4NxM0, TxN1-2M0, TxNxM1; G1-2; n=141), and stage IVb tumors were considered metastatic (T4NxM0, TxN1-2M0, TxNxM1; G3-4; n=2050). The 5-year CSS rates for stage IVa and IVb patients were 76.3% (95% CI 68.7% to 83.9%) and 31.4% (95% CI 28.5% to 34.3%), respectively (*P*<0.001).

**Conclusions:** Stage IV patients were stratified into two prognostically different risk groups depending on metastasis or grade. The subclassification of stage IV can increase the level of prognostic detail and individualize the prediction of survival in invasive UTUC patients.

## Introduction

Urothelial carcinomas are the fourth most common tumors 1. The American Joint Committee on Cancer/Union Internationale Contre le Cancer (AJCC/UICC) tumor-node-metastasis (TNM) staging system is widely used to predict upper tract urothelial carcinoma (UTUC) patient prognosis, guide treatment options, and evaluate treatment results from different centers. UTUCs are uncommon and account for only 5-10% of urothelial carcinomas; however, 60% of UTUCs progress to invasive disease 2. Because of the rarity of this type of cancer, relatively limited data regarding TNM staging have been obtained by the European Association of Urology (EAU) and the National Comprehensive Cancer Network (NCCN) 2, 3.

Several studies have verified the validity of the prognostic group classifications 2, 4-6. In 2017, the N2 and N3 classifications were redefined in the 8^th^ revision of the TNM staging system 7. However, the AJCC prognostic group classifications have remained almost unchanged; stage IV still includes T4, N+ and M1 7. Therefore room for improvement still exists in the AJCC prognostic group classifications 8, 9.

In this study, we critically analyzed the controversial areas of the AJCC-TNM prognostic group staging system to discuss whether the modified AJCC-TNM prognostic stage group IV can be subdivided to provide more detailed prognoses for patients with invasive UTUC.

## Materials and methods

Patients diagnosed with UTUC (ICD-O-2 C65.9 and C66.9 codes) with available TNM stage classification information between 2004 and 2015 were identified from 18 Surveillance, Epidemiology and End Results (SEER) registries. Data from Alaska; Atlanta; California, excluding San Francisco (SF)/San Jose/Monterey (SJM)/Los Angeles (LA); Connecticut; Detroit; Greater Georgia; Hawaii; Kentucky; Los Angeles; Rural Georgia; Louisiana; Iowa; New Jersey; New Mexico; San Francisco; San Jose; Seattle; and Utah were obtained. The characteristics of the SEER population are comparable to those of the general population of the United States.

**Figure 1:**
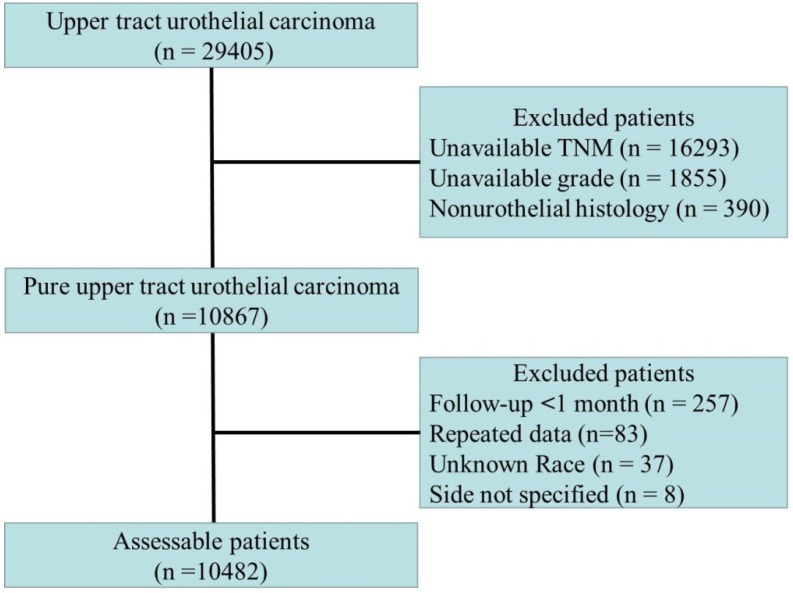
Flow chart of patients included in the primary analysis.

Among the included patients, only patients with transitional cell carcinoma or papillary transitional cell carcinoma confirmed by histology were considered. The inclusion and exclusion criteria are shown in **Fig. 1**. The cause of death (cancer-specific versus noncancer related) was defined according to the SEER assignment of the cause of death. Patients who did not die of UTUC were considered to have died due to other causes. The histopathological data were reviewed by an independent pathology committee, and all histopathological reports were based on the AJCC-TNM staging system 7. For repeated data, the highest level of histopathological data or prime data were considered.

The outcome of interest was cancer-specific survival (CSS). The Kaplan-Meier method was used to determine the CSS. The log-rank test was used to compare CSS rates between different groups. Statistical analyses were performed with Statistical Package for the Social Sciences software (SPSS version 23, IBM Corp, Armonk, NY). A two-sided *P* value less than 0.05 was considered statistically significant.

## Results

A total of 10,482 eligible patients were identified; 2428 patients (23.2%) died of UTUC, and 3295 patients (31.4%) died of other causes (2004 to 2015) (Table 1). There were 6251 men (59.6%) and 4231 women (40.4%) included in the study, with a median age of 74 yrs. Overall, 6418 (61.2%), 4051 (38.6%) and 13 (0.1%) patients had renal pelvic, ureteral and multifocal tumors, respectively. Lymph node removal was performed in 2711 patients (25.9%). The proportion of patients with nodal metastases was 13.5% (1414/10,482). The detailed clinicopathological characteristics of the cohort are listed in **Table 1**.

**Figure 2:**
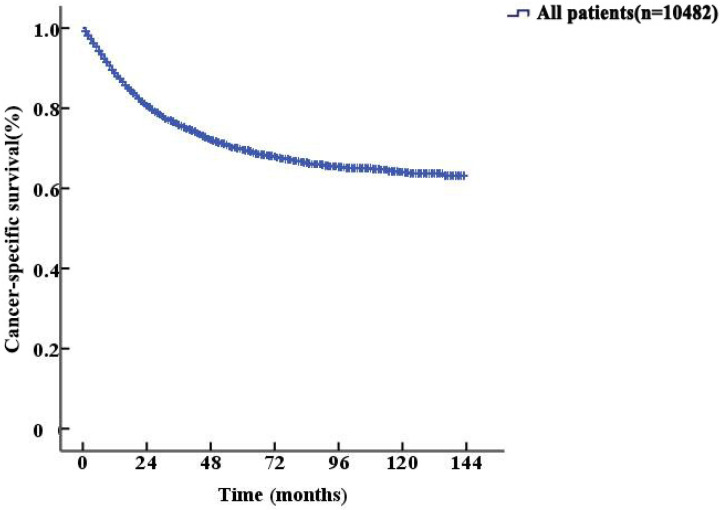
Kaplan-Meier cancer-specific survival (CSS) curves of patients with invasive upper tract urothelial carcinoma.

**Figure 3:**
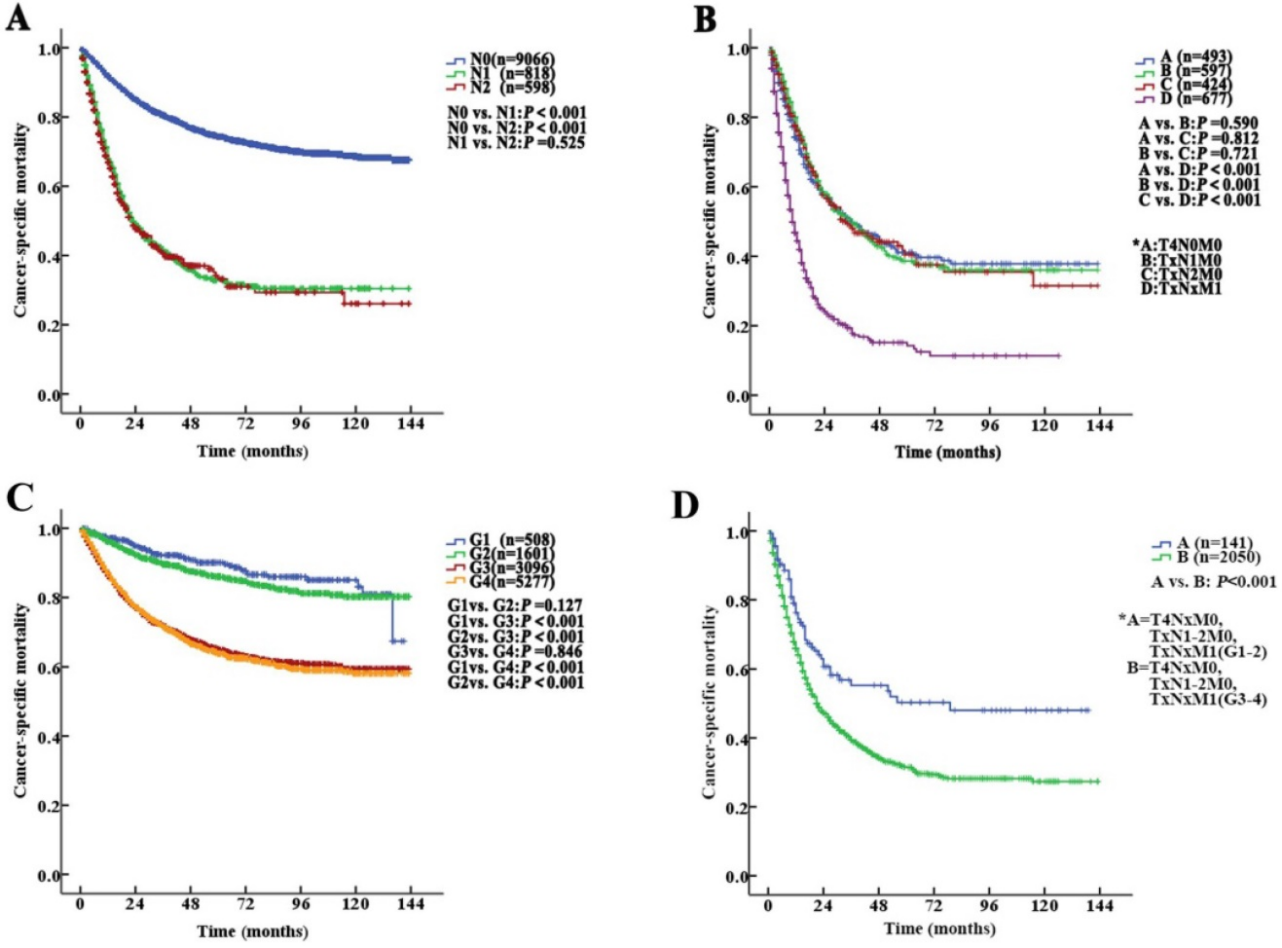
Kaplan-Meier cancer-specific survival (CSS) curves of patients with invasive upper tract urothelial carcinoma at different N stages and grades. A. Stratification by N stages, the 5-year CSS curves show no significant difference between patients with N1 and N2 disease. B. Stratification by stage IV of the AJCC-TNM prognostic group staging system with nonmetastatic or metastatic disease (A=T4N0M0; B=TxN1M0; C=TxN2M0 and D=TxNxM1). C. Stratification by grade, the 5-year CSS curves also show no significant difference between patients with G1 and G2 disease or between patients with G3 and G4 disease. D. Stratification by stage IV of the AJCC-TNM prognostic group staging system with low or high grade (A=T4NxM0, TxN1-2M0, TxNxM1; G1-2; B=T4NxM0, TxN1-2M0, TxNxM1; G3-4).

The median follow-up duration was 27 (1-143) months. The 5-year CSS of UTUC patients was 69.6% (68.4-70.8%). **Fig. 2** shows the CSS in UTUC patients. Mortality was significantly correlated with sex, race, tumor location, T stage, N stage, M stage, histologic grade and prognostic group (all *P*<0.001, **Table 2**). Interestingly, patients with N1 disease and N2 disease had similar mortality rates (5-year CSS: 32.8% vs. 33.2%, *P*=0.525, **Fig. 3A**). With regard to grade (G) classification, patients with G1 and G2 disease had similar mortality rates (*P*=0.127) to those of patients with G3 and G4 disease (*P*=0.846) (**Fig. 3B**).

**Figure 4:**
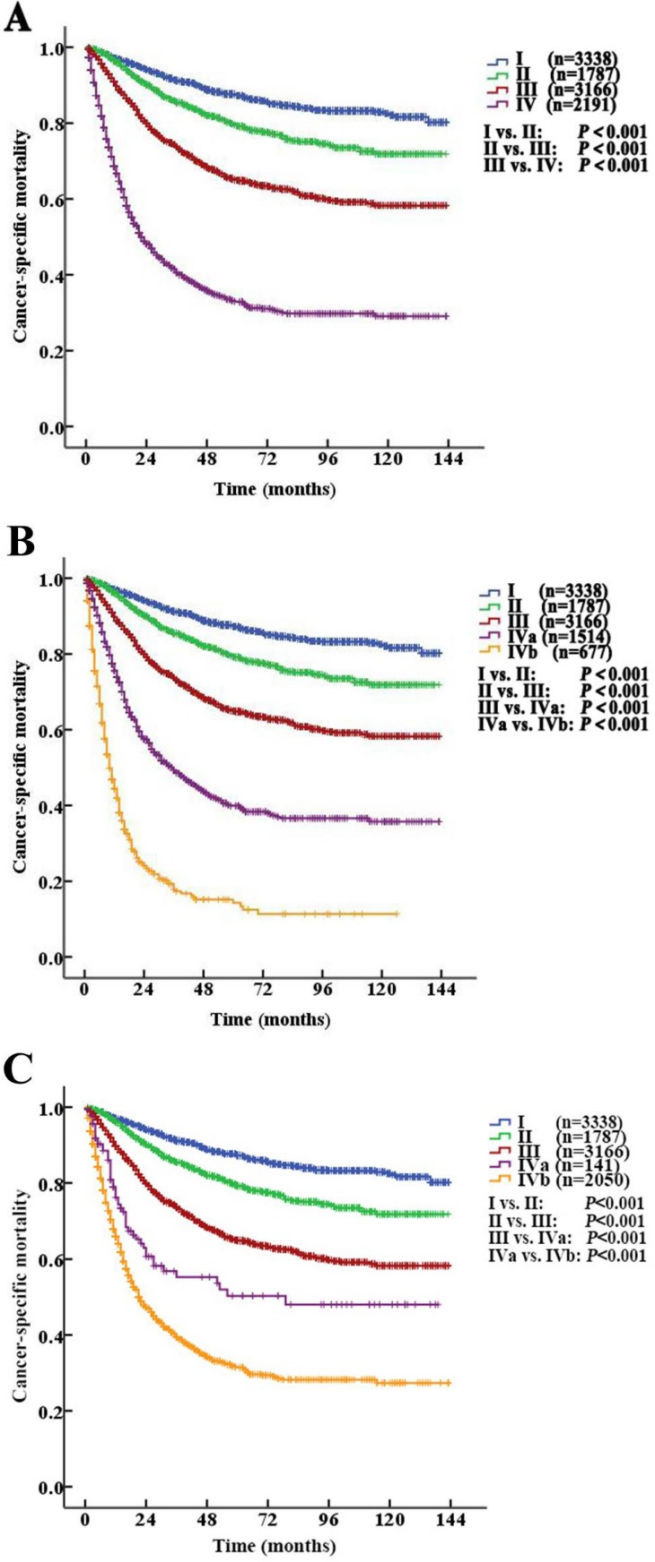
Kaplan-Meier cancer-specific survival (CSS) curves of patients with invasive upper tract urothelial carcinoma according to the AJCC-TNM prognostic group staging system. A. Stratification by the AJCC-TNM prognostic group staging system. B. Stratification by Modified Stage 1. IVa tumors were classified as nonmetastatic (T4NxM0 and TxN1-2M0), and stage IVb tumors were classified as metastatic (TxNxM1). C. Stratification by Modified Stage 2. IVa tumors were classified as low-grade (T4NxM0, TxN1-2M0, TxNxM1; G1-2), and stage IVb tumors were classified as high-grade (T4NxM0, TxN1-2M0, TxNxM1; G3-4).

According to the AJCC-TNM prognostic stage group criteria, the 5-year CSS rates were 87.3% (95% CI 85.9% to 88.7%), 79.3% (95% CI 76.9% to 81.7%), 64.9% (95% CI 62.7% to 67.1%) and 32.8% (95% CI 30.1% to 35.5%) in patients with stage I to IV disease, respectively (I *vs.* II, *P*<0.001; II *vs.* III, *P*<0.001; III *vs.* IV, *P* <0.001, *P*<0.001,** Fig. 4A**).

However, among the patients with stage IV disease, 493 (22.5%) had T4N0M0, 597 (27.2%) had TxN1M0, 424 (19.4%) had TxN2M0, and 677 (30.9%) had metastases (TxNxM1). Additionally, the mortality curves for the T4N0M0, TxN1M0 and TxN2M0 stage groups overlapped and crossed. The 5-year CSS rates for patients with stage IV disease classified as T4N0M0, TxN1M0, TxN2M0 and TxNxM1 were 41.1% (95% CI 35.2% to 47.0%), 38.6% (95% CI 33.1% to 44.1%), 40.4% (95% CI 33.0% to 47.8%) and 14.2% (95% CI 9.9% to 18.5%), respectively (T4N0M0 *vs.* TxNxM1, *P*<0.001; TxN1M0 *vs.* TxNxM1, *P*<0.001; TxN2M0 *vs.* TxNxM1, *P*<0.001, **Fig. 3C**). No significant differences in CSS were found among the patients with ≥2 site-specific metastases (n=73), single bone metastases (n=60), single lung metastases (n=89) and single liver metastases (n=45) (*P>0.10*).

Stage IV tumors were subdivided on the basis of mortality data (Modified Stage 1): stage IVa tumors were considered nonmetastatic (T4NxM0 and TxN1-2M0; n=1514), and stage IVb tumors were considered metastatic (TxNxM1, n=677). The 5-year CSS rates for patients with stage IVa and IVb disease were 39.9% (95% CI 36.4% to 43.4%) and 14.2% (95% CI 9.9% to 18.5%), respectively (*P* <0.001). These definitions provided improved prognostic stratification with significant differences in mortality between the modified categories (**Fig. 4B**).

Stage IV tumors were further subdivided (Modified Stage 2): stage IVa tumors were considered low grade (T4NxM0, TxN1-2M0, TxNxM1; G1-2; n=141), and stage IVb tumors were considered metastatic (T4NxM0, TxN1-2M0, TxNxM1; G3-4; n=2050). The 5-year CSS rates for patients with stage IVa and IVb disease were 76.3% (95% CI 68.7% to 83.9%) and 31.4% (95% CI 28.5% to 34.3%), respectively (*P*<0.001,** Fig. 3D**). These definitions provided improved prognostic stratification with significant differences in mortality between the modified categories (**Fig. 4C**).

Harrell's concordance index (C-index) and bootstrap-corrected C-index values for the stages are shown in** Table 3.** The value of the modifications did not decrease.

## Discussion

To enable prognostic prediction, the AJCC/UICC-TNM system was established using a mix of clinical and pathologic diagnoses, and it accurately reflects the prognosis in patients with UTUC 7. The TNM classification is widely used to determine prognosis and inform the selection of an effective treatment. In the current AJCC-TNM prognostic group staging system, stage IV is defined as tumor invasion of the adjacent organs or through the kidney into the perinephric fat, lymph node metastasis and distant metastasis 2, 7. However, the stage IV criteria are similar to those of previous versions without any recent changes 10, 11. In this study, we critically analyzed the heterogeneity of the stage IV criteria and discussed the predictive value and feasibility of a staging system for invasive UTUC. Based on different pathological factors, two modifications were proposed to help better differentiate AJCC stage IV disease without any loss of predictive value.

The prognosis of UTUC varies with stage. The extent of a tumor or metastasis is a confirmed prognostic factor 12, 13. Tumor stage is one of the most important predictors of survival in patients with UTUC 11, 14, 15. Additionally, the involvement of the lymph nodes in UTUC is an independent predictive factor for poor survival 16, 17. The EAU guidelines also affirm that postoperative factors, including tumor stage, lymph node involvement, and ureteral and/or multifocal tumors, affect prognosis 3. Our study showed that the 5-year CSS rates for patients with stage I, II, III and IV disease were 87.3% (95% CI 85.9% to 88.7%), 79.3% (95% CI 76.9% to 81.7%), 64.9% (95% CI 62.7% to 67.1%) and 32.8% (95% CI 30.1% to 35.5%), respectively (I *vs*. II, *P*<0.001; II *vs.* III, *P*<0.001; III *vs.* IV, *P*<0.001, all: *P*<0.001).

Tumor grade is also one of the most important predictors of survival in patients with UTUC 18. The updated World Health Organization Classification provides more useful insights into and approaches to individual categories 6, 19, 20. In our study, stage IV tumors were subdivided as follows (Modified Stage 2): stage IVa tumors were considered low grade (T4NxM0, TxN1-2M0, TxNxM1; G1-2), and stage IVb tumors were considered metastatic (T4NxM0, TxN1-2M0, TxNxM1; G3-4), and there was a significant difference in mortality.

UTUC shares a similar etiology and histologic distribution with urothelial cancers of the bladder 19. According to the AJCC prognostic stage for tumors of the urinary bladder, stage IV was subdivided into stage IVa (T4bN0M0 and TxNxM1a) and stage IVb (TxNxM1b) on the basis of the presence of distant metastases 21. Stage IV UTUC tumors also include T4N0M0, TxN1M0, TxN2M0 and TxNxM1. Interestingly, we found that the stage IV subgroups were heterogeneous. The mortality curves for T4N0M0, TxN1M0 and TxN2M0 overlapped and crossed. In addition, M1 disease was generally associated with a poor prognosis. Further analysis, divided stage IV upper urinary tract carcinomas into locoregionally advanced and metastatic disease subcategories. The molecular mechanisms underlying the observed interaction between locally advanced disease and distant metastases in UTUC patients may help clarify the results of this study 8.

There are few accurate predictive tools for UTUC 3. Nomograms, which use demographic and clinicopathologic data to predict oncologic outcomes, have attracted increased interest in the past few years 22-24. Nomogram models have been constructed using different factors, so there is no consensus yet, and they are not widely used in clinical practice. Currently, AJCC prognostic stage groups are used to discriminate between prognostic subgroups within a patient population 25. The treatment of UTUC differs significantly based on stage 26-28. Our study suggests a subgroup analysis is useful in the stage IV group to establish more individualized therapeutic strategies and prognostic predictions. It is conceivable that the inclusion of detailed pathologic information could further enhance the individuation of the current model.

We acknowledge that our study had some limitations. First, the analyses were retrospective in nature because the collection of data from the SEER database was retrospective. The treating physician's perception of the patient's prognosis and the treatment (especially adjuvant therapies) were not included in the data. Second, other variables may be beneficial for predicting CSS. Unfortunately, the upper urinary tract tumor location, tumor architecture, tumor size, presence of lymphovascular invasion, molecular biology (chromosome abnormalities), biomarkers, and lymph node dissection could not be obtained from the SEER database. We did not calculate the difference with regard to established modifications because some factors could not be collected. We compared the newly developed modifications with the 8th AJCC-TNM classification system. The predictive accuracy of the modified AJCC-TNM prognostic group staging system should be tested in an external cohort to determine its validity with regard to prediction in clinical practice. Third, we failed to contribute any original laboratory finding or clinical observation from patients for external verification. However, we believe that different established modifications could be verified with unified data. Thus, all analyses should be considered exploratory rather than hypothesis-driven. Despite these limitations, we believe that a simple analysis is important to establish a basis for future validation studies with larger multicenter datasets.

Notwithstanding the above limitations, the current AJCC-TNM prognostic group staging system has room for improvement. Stage IV was stratified into two prognostically different risk groups depending on metastasis or grade. Revisions to the current AJCC-TNM prognostic group staging system may make it more concise and provide more detailed information to patients with invasive UTUC.

## Figures and Tables

**Table 1:** Clinical and pathological characteristics of patients with upper tract urothelial carcinoma

Variable	Entire cohort (n=10482)
**Age (y)**	
Mean	72.3
Median	74.0
Range	22.0-101.0
**Sex**	
Male	6251 (59.6)
Female	4231 (40.4)
**Race**	
Caucasian	9230 (88.1)
Other	1252 (11.9)
**T stage**	
T1	3557 (33.9)
T2	1941 (18.5)
T3	3962 (37.8)
T4	1022 (9.8)
**N stage**	
N0	9066 (86.5)
N1	818 (7.8)
N2	598 (5.7)
**M stage**	
M0	9805 (93.5)
M1	677 (6.5)
**Grade**	
G1-2	2109 (20.1)
G3-4	8373 (79.8)
**Tumor location**	
Renal pelvis	6418 (61.2)
Ureter	4051 (38.6)
Both	13 (0.1)
**Laterality**	
Right	5166 (49.3)
Left	5307 (50.6)
Bilateral	9 (0.1)
**Prognostic groups**	
I	3338 (31.8)
II	1787 (17.0)
III	3166 (30.2)
IV	2191 (20.9)

**Table 2:** The 5-year cancer-specific mortality in patients with upper tract urothelial carcinoma

Variable	5-year CSS	*P* value
**Age (y)**		
Mean		
Median		
Range		
**Sex**		<0.001
Male	77.9 (70.7-73.5)	
Female	66.0 (64.2-67.8)	
Race		<0.001
Caucasian	70.5 (69.3-71.7)	
Other	63.1 (59.8-66.4)	
**T stage**		<0.001
T1	84.7 (83.3-86.1)	
T2	77.2 (74.8-79.6)	
T3	59.2 (67.2-61.2)	
T4	29.4 (25.3-33.5)	
**N stage**		<0.001
N0	74.4 (73.2-75.6)	
N1	32.8 (28.3-37.3)	
N2	33.2 (27.1-39.3)	
**M stage**		<0.001
M0	72.6 (71.4-73.8)	
M1	14.2 (9.9-18.5)	
**Grade**		<0.001
G1-2	87.0 (85.2-88.8)	
G3-4	64.4 (63.0-65.8	
Tumor location		<0.001
Renal pelvis	72.4 (70.6-74.2)	
Ureter	67.8 (67.6-78.0)	
Both		
**Laterality**		0.380
Right	68.9 (67.3-70.5)	
Left	70.3 (68.7-71.9)	
Bilateral		
**Prognostic groups**		<0.001
I	87.3 (85.9-88.7)	
II	79.3 (76.9-81.7)	
III	64.9 (62.7-67.1)	
IV	32.8 (30.1-35.5)	

**Table 3:** Predictive accuracy of the staging system

Stage	C-index	Bootstrap C-index
AJCC-TNM	0.73	0.72
Modification 1	0.74	0.74
Modification 2	0.73	0.73

## Abbreviations

AJCC/UICC: American Joint Committee on Cancer/Union Internationale Contre le Cancer
AUC: area under the curve
CSS: cancer-specific survival
EAU: European Association of Urology
NCCN: National Comprehensive Cancer Network
SEER: Surveillance, Epidemiology and End Results
TNM: tumor-node-metastasis
UTUC: upper tract urothelial carcinoma
